# Assessment of the implementation of accelerated drug marketing registration procedures for antineoplastic and immunomodulating agents in China: based on 2016–2022 review data

**DOI:** 10.3389/fphar.2024.1345672

**Published:** 2024-03-18

**Authors:** Yipeng Lan, Xiaofeng Lin, Jialin Yu, Li Wang, Lihua Sun, Zhe Huang

**Affiliations:** ^1^ School of Business Administration, Shenyang Pharmaceutical University, Shenyang, China; ^2^ Institute of Drug Regulatory Science, Shenyang Pharmaceutical University, Shenyang, China

**Keywords:** antineoplastic and immunomodulating agents, accelerated drug marketing registration procedures, drug review and approval, breakthrough therapy drug, conditional approval, priority review and approval, China

## Abstract

**Objective:**

Since 2016, China has successively implemented Accelerated Drug Marketing Registration Procedures (ADMRPs) for drugs, including Breakthrough Therapy Drug (BTD), Conditional Approval (CA), and Priority Review and Approval (PRA), which have played an important role in promoting the development and review of clinically urgently needed drugs. In this study, we focused on the antineoplastic and immunomodulating agents approved for marketing through ADMRPs, to provide a reference for promoting the formation of a stable and mature regulatory system for the review and approval of antineoplastic drugs and immunomodulating agents in China.

**Methods:**

Reviewed the National Medical Products Administration (NMPA) drug review reports for the years 2016–2022 and screened the antineoplastic and immunomodulating agents approved through ADMRPs. Then, with the help of the NMPA website and the Yaozhi Database, two researchers independently queried and entered the detailed information of the selected drugs, and checked with each other. The attribute classification and main characteristics of the drugs were then analyzed with descriptive statistics to obtain the trend of drug types, drug review and approval status, and timeliness.

**Results:**

A total of 206 antineoplastic and immunomodulating agents were approved for marketing through five accelerated marketing registration procedures (or procedure combinations), with the average review time shortened by about 81 days. Among them, imported drugs accounted for a larger proportion, the most drugs for treating non-small cell lung cancer and lymphoma, and the largest number of PD-1/PDL-1 inhibitors, but pediatric drugs and rare disease drugs accounted for a smaller proportion.

**Conclusion:**

ADMRPs can promote the accessibility of antineoplastic and immunomodulating agents in China and safeguard the life and health rights of more patients. Nevertheless, it is necessary to pay attention to the expansion of the types of indications for medicines and to increase the development of drugs that are urgently needed by a small number of patients.

## 1 Introduction

As early as 1996, the World Health Organization (WHO) included antineoplastic and immunomodulating agents into the same category in the Anatomical Therapeutic Chemical (ATC) system for management based on the anatomical classification of cancer and immune diseases and the pathogenesis of the diseases (Furlow, 2021; Mikhael et al., 2021). Since then, the development of antineoplastic and immunologic drugs has attracted the attention of the global pharmaceutical industry. In recent years, with the aging of the population and changes in the disease spectrum, there has been a trend towards an increased burden of cancer and immunization diseases globally, especially in developing countries. 19.29 million new cancer cases occurred globally in 2020, and it is expected that the burden of cancer will increase by 50 percent by 2040 when the number of new cancer cases worldwide will reach nearly 30 million (Bray et al., 2021; Sung et al., 2021). In addition, according to a research study, the prevalence of immune diseases increased rapidly from 7.7% in 2000–2002 to 11.0% in 2017–2019 (Conrad et al., 2023). China has a large population base with 1.41 billion people. In 2020, China had 4.57 million new cancer cases, accounting for 23.7% of the world’s total, with the number of new cancer cases far exceeding that of the rest of the world, and the prevalence of immune diseases also showed an increasing trend (Xia et al., 2022; Chen et al., 2023). In 2006, the WHO explicitly classified oncological diseases and some immune diseases as chronic diseases, and many countries subsequently formulated relevant measures to deal with these diseases (Yabroff et al., 2011; Luengo-Fernandez et al., 2013; Ward et al., 2021; Cao et al., 2022). In recent years, as tumor treatment has moved from the era of cytotoxic drug therapy to the era of targeted therapy and immunotherapy based on cytogenetics, molecular biology, and immunology, and the quality of patients’ lives has been effectively improved, the development of antineoplastic drugs and immunological drugs has become an important area of development for cross-fusion (Emens et al., 2017; Caini et al., 2022; Li et al., 2022; Pasetto and Lu, 2023; Swain et al., 2023). It has become a global consensus to accelerate the research and development (R&D) and approval of antineoplastic and immunomodulating agents to meet the demand for drugs for clinical use in related diseases (Fu et al., 2022; Zhang et al., 2022).

China is the second largest prescription drug market in the world, and with the rising incidence of oncology and immune diseases, the demand for antineoplastic and immunomodulating agents is growing (Huang et al., 2021; Zhong et al., 2021; Shang et al., 2023). To this end, China’s National Medical Products Administration (NMPA) has carried out a series of reforms to the drug review and approval system since 2015, as shown in Supplementary Table S1, which has had a huge impact on the R&D, approval, and clinical application of antineoplastic and immunomodulating agents. In particular, the successive implementation of the four Accelerated Drug Marketing Registration Procedures (ADMRPs), namely the Priority Review and Approval (PRA), the Conditional Approval (CA), the Breakthrough Therapy Drug (BTD), and the Special Approval (SA) has led to the accelerated approval and marketing of more clinically urgently needed drugs (National Medical Products Administration, 2020). The scope of application and application procedures for the four ADMRPs are shown in Supplementary Table S2. In addition, the “Healthy China Action - Cancer Prevention and Control Implementation Plan (2019–2022)" was jointly formulated by the National Health Commission and other agencies in 2019, encouraged the R&D and accelerated approval of antineoplastic and immunomodulating agents within the country, and facilitated the simultaneous marketing of new drugs from abroad in China, to achieve the enhancement of the accessibility of antineoplastic and immunomodulating agents. (National Health Commission, 2019).

In this paper, we analyzed the trend of approved drug types, drug review, and approval situation, and timeliness based on the data of the annual review report of the Center for Drug Evaluation (CDE) of NMPA for the period of 2016–2022, combing the information related to antineoplastic and immunomodulating agents approved through ADMRPs, including the drug name, incorporation procedures, source (domestic or imported), drug mechanism of action, target and indication, etc., to provide references to promote the review and approval of China’s antineoplastic and immunomodulating agents to form a stable and mature regulatory system.

## 2 Materials and methods

In the pre-study period, we learned that the CDE released the 2022 Annual Drug Review Report on 6 September 2023. Meanwhile, considering that China has successively implemented the ADMRPs since 2016, we selected the 2016–2022 Annual Drug Review Report as the main reference for obtaining the drugs approved for marketing through the ADMRPs. Then, the NMPA public data (https://www.nmpa.gov.cn/) and the Yaozhi Database (https://db.yaozh.com/) were used as the main data sources to obtain more drug attributes and review information, including acceptance number, the manufacturer, the accelerated marketing registration procedures including, the ATC classification, the indication, the target, the date of filing, the date of approval, and other information, which were entered into EXCEL 2022. This data entry process was initiated on 1 October 2023, and was completed independently and cross-checked by two researchers, with the research team members consulting together if they encountered disagreements. In the process, we found some drugs with different acceptance numbers because they had different specifications. However, according to the relevant descriptions in the Drug Review Reports issued by CDE and the practice in several literature, the same drug with different specifications declared by the same company at the same time was recognized as one (Chen et al., 2022; Luo et al., 2023; Su et al., 2023). Therefore, in this paper, the statistics were also conducted in this way.

After 2 weeks, all the data were entered and checked for accuracy, and antineoplastic and immunomodulating agents were screened according to the ATC classification. Then the data included in the study were analyzed by descriptive statistics using EXCEL 2022 and graphing using Origin 2019b to derive the trend of drug classes, drug review and approval, and timeliness of antineoplastic and immunomodulating agents approved for marketing by adopting the ADMRPs.

## 3 Results

### 3.1 Analysis of the number of approvals and inclusion procedures

**FIGURE 1:**
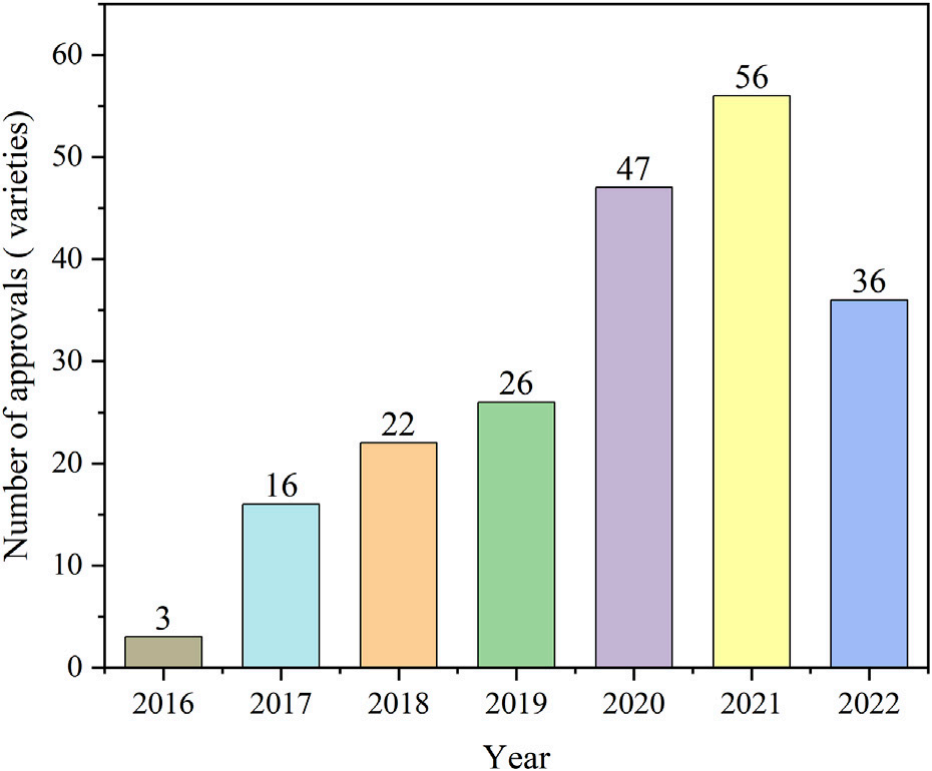
Number of antineoplastic and immunomodulating agents approved through the accelerated marketing registration procedures in 2016–2022.

**FIGURE 2:**
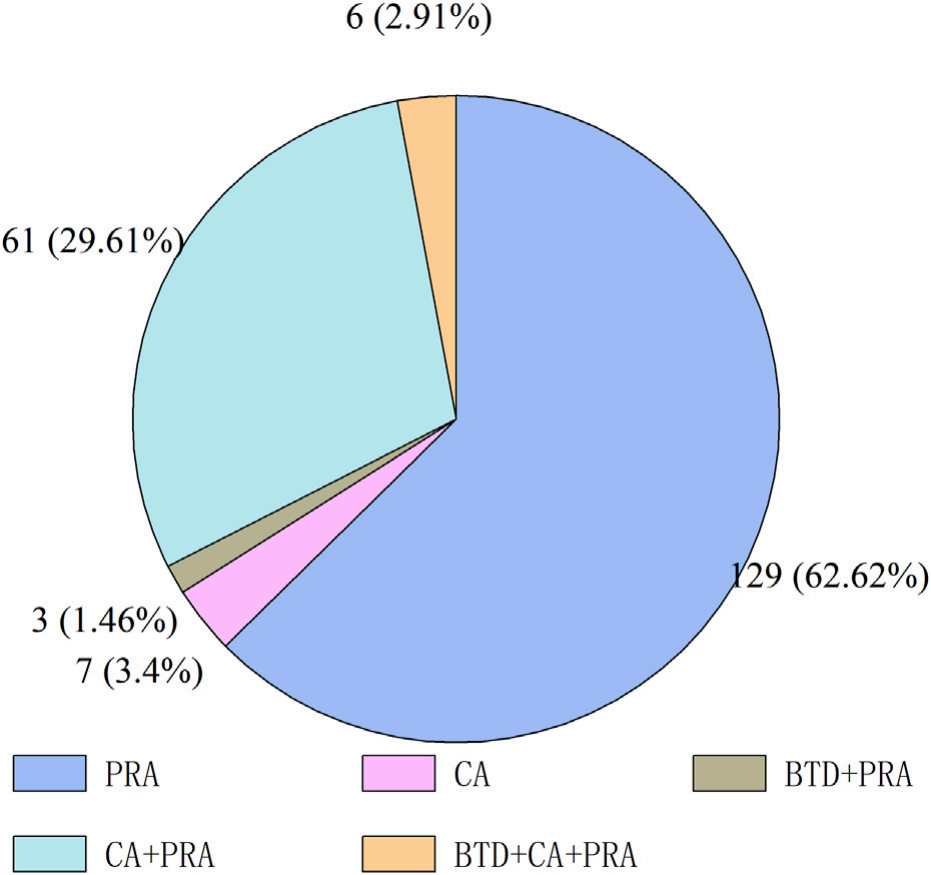
Accelerated drug marketing registration procedures adopted for approved marketed antineoplastic and immunomodulating agents.

Figure 1 shows the number of antineoplastic and immunomodulating agents approved through ADMRPs from 2016 to 2022. As can be seen from the figure, in recent years, the Chinese government increased its attention to the review and approval of antineoplastic and immunomodulatory drugs, and the number of drugs approved for marketing increased year by year, with the largest number of approvals in 2021 at 56. The number in 2022 decreased, which was mainly attributed to the phenomenon of market saturation of some antineoplastic and immunomodulating agents, as well as the impact of the COVID-19 epidemic on the development review and approval of drugs (Su et al., 2020), but still more than the number before the epidemic (in 2019). Among the 206 approved drugs, five main procedures (or procedure combinations) were adopted, as shown in Figure 2. PRA procedures were adopted most, with 129 products, accounting for 62.62%; followed by the combination of CA + PRA procedures, with 61 products, accounting for 29.61%. The other three were CA (3.4%), BTD + PRA (1.46%), and BTD + CA + PRA (2.91%). The reason for the combination of two or three procedures is that, for varieties included in the BTD, the applicant may apply for CA and an application for PRA when applying for marketing authorization for the drug if the applicant is assessed to comply with the relevant conditions; the applicant may also apply for the adoption of a PRA for the drug included in the CA.

### 3.2 Quantitative analysis of drugs imported

**FIGURE 3:**
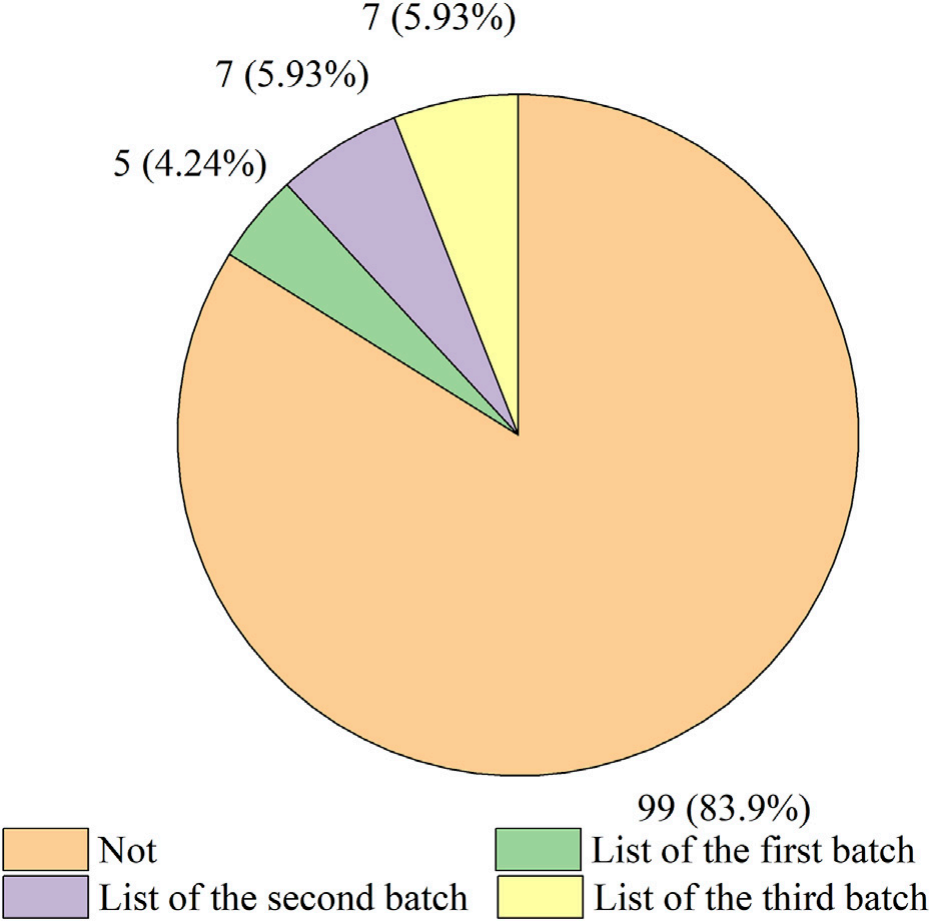
Distribution of the number of overseas antineoplastic and immunomodulating agents in urgent clinical need.

Of all the drugs, 116 antineoplastic and immunomodulating agents from overseas had been approved to be marketed in China through ADMRPs, accounting for 57.14%, while domestically produced drugs accounted for only 42.86%. Some of the imported drugs had also benefited from the preferential policies promulgated by the Chinese government to enter the Chinese market through the List of Overseas New Drugs Urgently Needed in Clinical Settings. In November 2018, the CDE released the List of the First Batch of Overseas New Drugs Urgently Needed in Clinical Settings, and as of November 2022, three batches of the list had been released, with a cumulative total of 73 drugs included (Center for Drug Evaluation, 2018; Center for Drug Evaluation, 2019; Center for Drug Evaluation, 2020). According to Figure 3, a total of 19 varieties were included in the list of 116 imported drugs counted. Among them, five varieties were included in the “List of the First Batch of Overseas New Drugs Urgently Needed in Clinical Settings”, namely: Secukinumab Injection, Pembrolizumab Injection (2), Olaparib, Dinutuximab beta Injection. Seven varieties were included in the “List of the Second Batch of Overseas New Drugs Urgently Needed in Clinical Settings”, namely: Adalimumab Injection (3), Apalutamide, Olaparib, and Pembrolizumab Injection (2). Another seven varieties were included in the “List of the Third Batch of Overseas New Drugs Urgently Needed in Clinical Settings”, namely: Teriflunomide, Palbociclib Capsules, Olaparib, Alectinib Hydrochloride Capsules, Pembrolizumab Injection, Dimethyl Fumarate Enteric Capsules, and Giritinib Fumarate Tablets. The above drugs included in the list were included by CDE in PRA to accelerate the review, which to a certain extent solved the dilemma of drugs available outside but not available within China, and fulfilled the clinical drug needs of some patients.

### 3.3 Distribution of rare disease drugs and pediatric drugs

Unlike the United States, which has an Orphan Drug Act specifically for rare diseases, China has not yet introduced a bill on rare disease certification. As to what constitutes a rare disease, it is mainly based on the Rare Disease Catalog issued by the National Health Commission as the basis for identification. As of October 2023, the National Health Commission had released two batches of Rare Disease Catalogs, including 207 diseases (National Health Commission, 2018; National Health Commission, 2023). The Second Batch of the Rare Disease Catalog was released in September 2023, which was outside the timeframe of our research data. Therefore, only the First Batch of Rare Disease Catalog was used as a reference. As can be seen from Table 1, among these antineoplastic and immunomodulating agents, there were only 10 drugs belonging to the same category of rare disease drugs, accounting for 4.85%. Accordingly, China still has a long way to go in strengthening the management of rare diseases, guaranteeing the accessibility of medicines for patients with rare diseases, and accelerating the development and approval of antineoplastic and immunomodulatory drugs for rare diseases.

**TABLE 1:** Distribution of the number of drugs for rare diseases and children among antineoplastic and immunomodulating agents.

	Yes	Not
Drugs for rare diseases	10 (4.85%)	196 (95.15%)
Drugs for children	11 (5.34%)	195 (94.66%)

In addition, as shown in Table 1, only 11 antineoplastic and immunomodulating agents for children, accounted for 5.34% of the medicines included in the analysis. Although the proportion of children suffering from cancer or immune diseases is not as high as that of adults, the demand for children’s antineoplastic and immunomodulatory drugs in China is higher than that of other countries. At present, there are few types of medicines for children on the market and a single dosage form, so it is also necessary to pay attention to the development and approval of antineoplastic and immunomodulatory drugs used by children, to improve the accessibility of medicines for children.

### 3.4 Distribution of indications and targets of drugs

**FIGURE 4:**
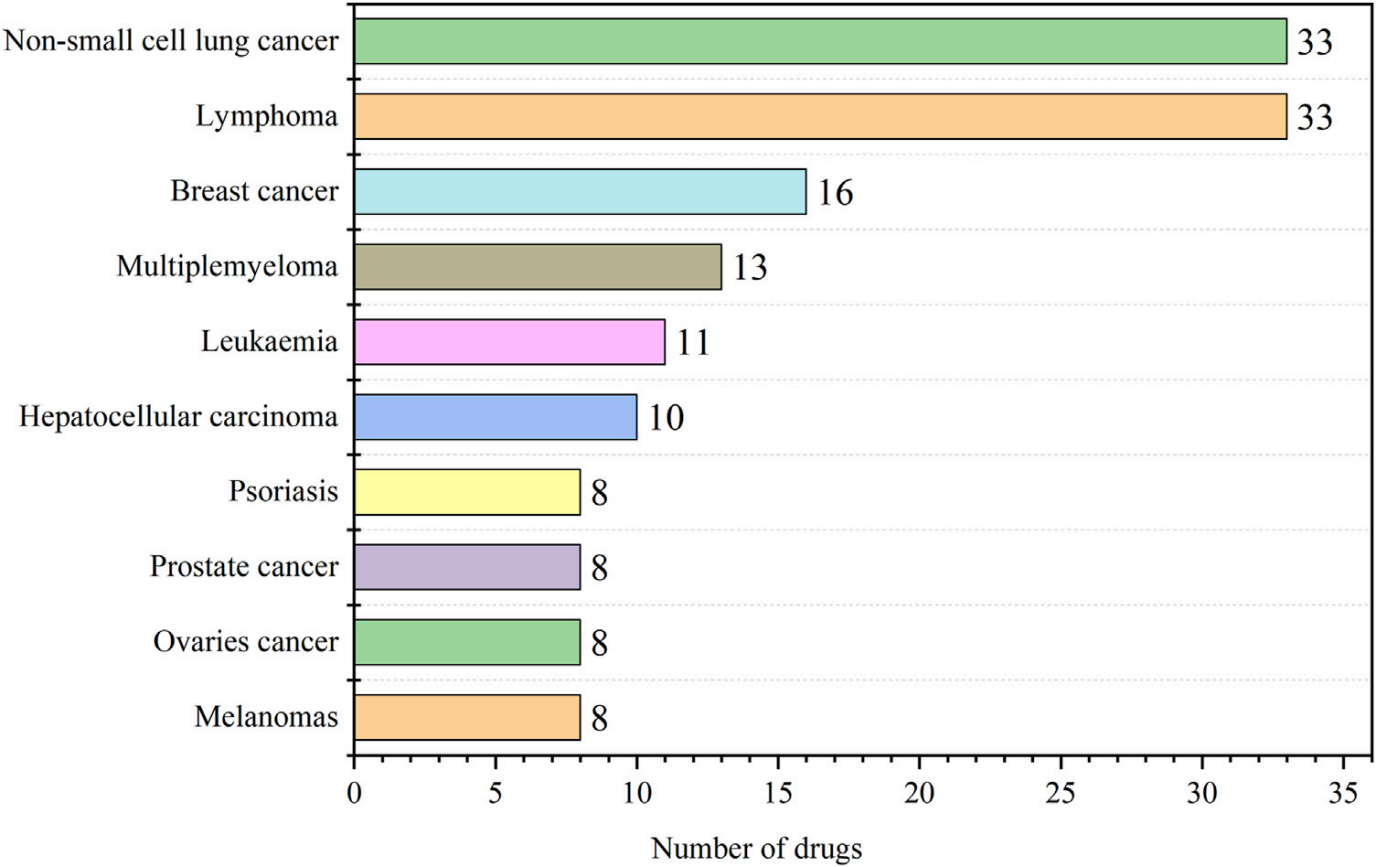
Distribution of indications for antineoplastic and immunomodulating agents (top 10).

According to the data feedback, the 206 indications of antineoplastic and immunomodulating agents that were included contained 44 diseases, and the top ten indications in terms of the number were summarized and counted as shown in Figure 4, and all the indications and the corresponding number of drugs are detailed in the Supplementary Table S3. Of all the drugs, the number of anticancer drugs was predominant, with non-small cell lung cancer and lymphoma having the highest number at 33. Lung cancer is one of the malignant tumors with high morbidity and mortality rates worldwide. Lung cancer ranked first among all new cases of malignant tumors in China in 2022, accounting for 18.06%, and the number of deaths due to lung cancer accounted for 23.9% of the total number of deaths due to malignant tumors in China, which likewise ranked first. The analysis of the data on approved drug indications and the current distribution of cancer in the Chinese population showed that the R&D and approval of antitumor drugs in China were matched with the situation of cancer patients.

Further, according to the mechanism of action and type of target of the drugs, the classification and quantity statistics were made, and the contents shown in Table 2 were obtained. According to the action type of drugs, they can be divided into four major categories: antitumor drugs, immune-promoting drugs, immune-suppressive drugs, and endocrine therapies, among which antitumor drugs were the most numerous. In terms of drug mechanism of action, the largest number of monoclonal antibodies and antibody-drug couplings was found; while in terms of target type, the largest number of Programmed Cell Death Protein-1/Programmed Cell Death Protein Ligand-1 (PD-1/PDL-1) inhibitors was found, with 31.

**TABLE 2:** Classification statistics of antineoplastic and immunomodulating agents by mechanism of action and target type.

Type of action	Drug mechanism	Target type	Number (types)
Antitumor drugs	Antimetabolite	Pyrimidine homologs	2
		Folate congeners	3
	Monoclonal antibodies and antibody-drug conjugates	CD20 inhibitors	3
		CD22 inhibitors	1
		CD38 inhibitors	3
		HER2 inhibitors	4
		PD-1/PDL-1 inhibitors	31
		Trop-2 inhibitors	1
		VEGF/VEGFR inhibitors	3
		Epidermal growth factor receptor tyrosine kinase inhibitors (EGFR-TKIs)	19
		Other monoclonal antibodies and antibody-drug conjugates	13
	Protein kinase inhibitor	B-Raf serine-threonine kinase (BRAF) inhibitor	4
		Bruton tyrosine kinase (BTK) inhibitor	6
		Anaplastic lymphoma kinase (ALK) inhibitors	4
		Phosphatidylinositol-3-kinase (Pi3K) inhibitor	2
		Mitogen-activated extracellular signal-regulated kinase (MEK) inhibitors	3
		Cyclin-dependent kinase (CDK) inhibitors	3
		Other protein kinase inhibitors	20
	Other antitumor drugs	Hedgehog pathway inhibitors	2
		Proteasome inhibitors	3
		Poly (ADP-ribose) polymerase (PARP) inhibitors	8
		Tyrosine-protein kinase inhibitor	2
		Other antitumor drugs	11
	Alkylating agents	nitrogen mustard	5
	Cytotoxic antibiotics and related drugs	Anthracycline antibiotics and related drugs	1
		Other cytotoxic antibiotics	1
	Plant alkaloids and other natural medicines	Paclitaxel alkaloids	4
Immune-promoting drugs	immune-promoting drug	interferon drugs	1
		Other immune enhancers	2
Immune-suppressive drugs	immunosuppressant	Interleukin Inhibitors	5
		Calcium-modulated phosphatase inhibitor	1
		Selective immunosuppression	11
		Tumor necrosis factor-alpha (TNF-α) inhibitors	8
		Other immunosuppressants	7
Endocrine therapy	Hormonal antagonists and related drugs	Aromatase inhibitors	1
		Antiestrogenic drugs	1
		Antiandrogenic drugs	6
		Other hormone antagonists and related drugs	1

### 3.5 Statistics on the length of time for drug approval

According to a previous study, since the reform of the drug review and approval system in 2015, the average review time for new drug applications on the market was 483 days (Su et al., 2020). We calculated the evaluation time of antineoplastic and immunomodulating agents that adopted ADMRPs. The average evaluation time was about 402 days, which was 81 days shorter, and this was still in the case of the COVID-19 epidemic that had obstructed the review and approval of drugs. It can be seen that ADMRPs significantly shortened the review time of drugs and achieved the effects foreseen in the policy formulation.

**FIGURE 5:**
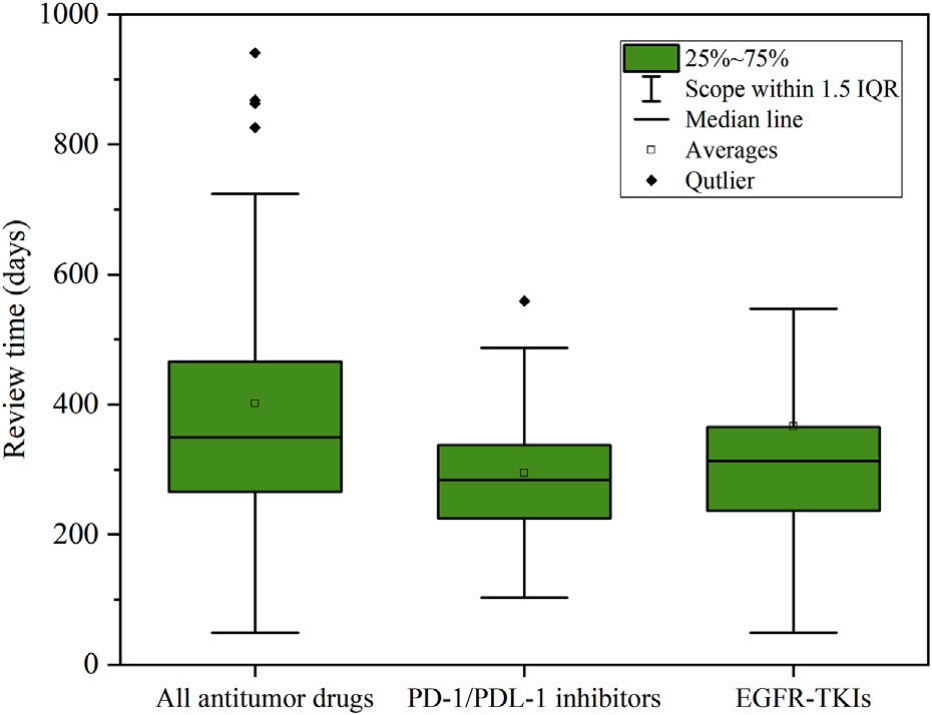
Statistics on the length of drug review.

In addition, we paid special attention to two hot areas in antitumor drugs: PD-1/PDL-1 inhibitors, as well as Epidermal Growth Factor Receptor Tyrosine Kinase Inhibitors (EGFR-TKIs). These two classes of antitumor drugs were not only hot areas of development in recent years, but also drugs with high clinical demand. As shown in Figure 5, the average review cycle for PD-1/PDL-1 inhibitors was about 295 days, which was 107 days shorter than the average review cycle for all drugs (*p* < 0.01);. In comparison, the average review cycle for EGFR-TKIs was 366 days, which was 36 days shorter (*p* < 0.01). This indicated that in the actual evaluation work, the CDE promoted the review and approval of drugs through comprehensive consideration oriented to the clinical needs and value of antitumor drugs, as well as the data reported by pharmaceutical companies, to meet the needs of a greater number of patients for therapeutic drugs.

## 4 Discussion

### 4.1 Opportunities for clinical use of antineoplastic and immunomodulating agents due to accelerated drug marketing registration procedures

The above results showed that more and more antineoplastic and immunomodulating agents were approved for marketing through ADMRPs. On the one hand, ADMRPs can prioritize the allocation of review resources. By meeting the needs of enterprises to communicate with drug regulatory authorities and shortening the review timeframe to accelerate the listing of drugs, it stimulated the enthusiasm of enterprises to carry out the research and development of antineoplastic and immunomodulating agents and improved the quality of the creation of products. On the other hand, some of the clinically needed antineoplastic drugs and immunomodulating agents can be put into clinical application more quickly to increase the choice of treatments and safeguard the public’s accessibility to medicines.

At the same time, this also required the applicant to conduct more rigorously designed post-marketing clinical research with more clinically valuable outcomes, to confirm the long-term clinical benefits and long-term safety outcomes of the drug for patients, and to improve the clinical data chain. In the case of CA, there were 68 drugs approved for marketing through CA (or CA process combinations). It should be emphasized that CA is based on alternative endpoints, intermediate clinical endpoints, or early clinical trial data, and applies to accelerating the listing of urgently needed drugs with outstanding clinical value in the form of “approval before validation” when complete clinical studies have not yet been completed, aiming to shorten the time of clinical research and development of the drug, and to bring advantageous products to the market as soon as possible with the “conditions”. Therefore, from the point of view of ensuring the safety of patients, the applicant must formulate a complete post-marketing risk management plan, to clarify the existing or identified risks and potential risks, and strengthen the post-marketing safety monitoring and risk control of the drug according to the plan (Nicolo et al., 2022).

### 4.2 Expansion of indications and scenarios for drugs approved for marketing through accelerated drug marketing registration procedures

From the number of approved drugs and indications, it was found that the currently approved indications of anticancer drugs were mainly focused on lung cancer, lymphoma, and breast cancer. Still, the data also revealed that fewer drugs had been approved in the fields of gastric cancer, esophageal cancer, uroepithelial cancer, metastatic colorectal cancer, cervical cancer, renal cell carcinoma, and other disease areas. These data reflected that the current development and review of antitumor drugs in China as a whole was in line with the order of the number of cancer cases in China, and more and more cancer patients had drugs available. However, China has a huge population base, and some of the cancer conditions with a small number of patients also have more than ten thousand people. For example, in 2022, the number of renal cell carcinoma patients in China was nearly 73,700 (Han et al., 2024), yet only one drug for the treatment of renal cell carcinoma had been approved for marketing through the Priority Review and Approval, which was unable to meet the patients’ demand for medication. Thus, enterprises need to make full use of ADMRPs to promote the development and declaration of anticancer drugs, and pay attention to the development of drugs for cancer diseases with fewer patients and the expansion of drug indications; government departments also need to publicize the convenience of ADMRPs through the feedback of the review data and provide technical guidance and support to eligible enterprises.

In addition, among the antineoplastic and immunomodulating agents approved through ADMRPs, the number of drugs for rare diseases or children was very small, at around 5%. Although PRA specified that new varieties, new dosage forms, and new specifications of medicines that meet the requirements of medicines for rare diseases or children with physiological characteristics of children could apply for the use of PRA, the effect of the policy’s actual operation was not very obvious. On the one hand, it was related to the fact that China paid less attention to children’s drugs and drugs for rare diseases in antineoplastic and immunomodulating agents, resulting in fewer approved, and on the other hand, it was also related to the fact that it was difficult to obtain subjects for clinical trials of children’s drugs and drugs for rare diseases. However, with the increased attention paid by the Chinese government to the use of drugs for children and rare diseases in recent years, coupled with the development of real-world research, more attention and support will be given to children’s antitumor drugs and rare diseases in ADMRPs in the future.

### 4.3 Strengthening post-marketing surveillance of drugs and establishing a multidimensional evaluation system for the value of antineoplastic and immunomodulating agents

For antineoplastic and immunomodulating agents approved for marketing through ADMRPs, strengthening post-marketing surveillance is an indispensable task to ensure the safety of medicines for patients. From the analysis of the review reports of the 68 drugs that adopted CA in this study, 84% completed phase II clinical trials, and a small number completed phase III clinical trials. Due to the limitations of clinical research is a challenge for the regulatory authorities to weigh the evaluation standards and control the risk-benefit, for drugs approved for marketing by adopting ADMRPs, to protect the urgent clinical needs and shorten the time to market of the drug at the same time, it should be better to do a good job of post-marketing supervision, and to consolidate the closed-loop management. CDE drafted the “Working Procedures for Approval of Application for Conditional Approval for Marketing of Drugs (for Trial Implementation) (Revised Draft for Public Comments)" in August 2023, which explicitly proposed the need for companies to submit clinical research progress reports annually, to quickly grasp the clinical benefits and risks of the drugs after their marketing and indirectly promote the research process (General Department of NMPA, 2023).

From the analysis results, it can be seen that the current developed and approved antitumor drugs were mainly concentrated in the field of higher economic value of the market, focusing on the phenomenon of popular targets was obvious, and there was a general phenomenon of homogenization of innovation, with PD1/PDL-1 being the most prominent. However, value-based health decision-making is influenced by multiple factors, and evaluating the value of antitumor drugs solely from an economic perspective to guide drug development and review is often one-sided. In addition to their high clinical value, antineoplastic drugs also have higher innovation value and social value than ordinary drugs, so it is particularly important to build a multidimensional drug value evaluation system to achieve a comprehensive evaluation of drugs. In 2021, the National Health Commission issued the Management Guidelines for Comprehensive Clinical Evaluation of Drugs, which had more important guiding significance for developing and clinical use guidance of antineoplastic and immunomodulating agents (National Health Commission, 2021). In the future, the idea of Multi-Criteria Decision Analysis (MCDA) can be used to develop a value evaluation system for antineoplastic and immunomodulating agents suitable for China and applied to the reform of the marketing approval system to clarify the screening criteria for accelerating the approval process for innovative drugs such as new antineoplastic and immunomodulating agents, providing a basis for accelerating the marketing of new antineoplastic and immunomodulating agents.

## 5 Conclusion

ADMRPs can significantly reduce the review time of antineoplastic and immunomodulating agents, promote the accessibility of these drugs in China, and promote the listing of clinically needed domestic or imported drugs in China, safeguarding patients’ rights and interests in life and health. However, in the future, it is also necessary to increase attention to the use of drugs for rare diseases and children in antineoplastic and immunomodulating agents and pay attention to the expansion of drug indications. In addition, it is necessary to explore establishing a system for evaluating the value of antineoplastic and immunomodulating agents, to provide a basis for accelerating the marketing of new drugs.

## Data Availability

The original contributions presented in the study are included in the article/Supplementary Material, further inquiries can be directed to the corresponding authors.
