# Supplementary material for: Assessment of the implementation of accelerated drug marketing registration procedures for antineoplastic and immunomodulating agents in China: based on 2016–2022 review data
Source: Front Pharmacol. 2024 Mar 18;15:1345672. doi: 10.3389/fphar.2024.1345672 (PMC10982497; doi:10.3389/fphar.2024.1345672)
Supplement: Supplementary file 1 [file Table1.DOCX]

Supplementary Material

# Supplementary Tables

**Supplementary** **Table 1** China's policy on accelerated drug review and approval

| Policy name | Release time | Accelerated drug review and approval related content |
| --- | --- | --- |
| Opinions of the State Council on Reforming the Review and Approval System for Drugs and Medical Devices | 2015 | Encourage clinical value-oriented drug innovation, optimize the review and approval procedures for innovative drugs, and expedite the review of innovative drugs in urgent clinical need. |
| Opinions on Resolving the Backlog of Drug Registration Applications and Implementing Priority Review and Approval | 2016 | Provide the scope, procedures, and work requirements for priority review and approval. |
| Opinions on Deepening the Reform of the Review and Approval System to Encourage Drug and Medical Device Innovation | 2017 | Drugs that are urgently needed for clinical purposes may be approved for marketing with conditions. |
| Drug Administration Law of the People's Republic of China | 2019 | Establishment of a series of management systems such as conditional approval, priority review and approval, and requirements to improve the drug review and approval work system, optimize the review and approval process and improve the efficiency of review and approval. |
| Provisions for Drug Registration | 2020 | Dedicated chapter on accelerated drug marketing registration procedures. |
| Working Procedures for the Review of Breakthrough Therapy Drugs (Trial) | 2020 | Provide for the scope of application, conditions, and review procedures for the review of breakthrough therapy drugs. |
| Working Procedures for the Review and Approval of Applications for Conditional Approval for Marketing of Drugs ( Trial ) | 2020 | Provide for the scope of application of the conditional approval process, its conditions, and the review process. |
| Working Procedures for Priority Review and Approval of Drug Marketing Authorization ( Trial ) | 2020 | Provide for the scope of application, conditions, and review and approval work procedures for priority review and approval work procedures. |

**Supplementary** **Table 2** Comparison of four accelerated drug marketing registration procedures in China

|  | Breakthrough therapy drug procedure (BTD) | Conditional approval procedure (CA) | Priority review and approval (PRA) | Special approval procedure (SA) |
| --- | --- | --- | --- | --- |
| Relevant legislation and policies | Article 59-62 of the Provisions for Drug Registration; Working Procedures for the Review of Breakthrough Therapy Drugs (Trial). | Article 26 of the Drug Administration Law; article 20 of the Vaccine Administration Law; articles 63-67 of the Provisions for Drug Registration; Working Procedures for the Review and Approval of Applications for Conditional Approval for Marketing of Drugs ( Trial ); and Technical Guiding Principles for Conditional Approval of Pharmaceuticals for Marketing ( Trial). | Article 16 of the Drug Administration Law; article 19 of the Vaccine Administration Law; articles 68-71 of the Provisions for Drug Registration; Working Procedures for Priority Review and Approval of Drug Marketing Authorization ( Trial ). | Articles 72-75 of the Measures for the Administration of Drug Registration; Procedures for Special Approval of Drugs. |
| Scope of application | Innovative or improved new drugs that are used to prevent or treat serious life-threatening diseases or diseases that seriously affect the quality of life, and for which there is no effective means of prevention or treatment, or for which there is sufficient evidence of a clear clinical advantage over existing treatments. | (1) Drugs for the treatment of serious life-threatening diseases for which there is no effective means of treatment, where data from clinical trials of the drugs are available to confirm the efficacy of the drugs and predict their clinical value; (2) Drugs urgently needed for public health, where data from clinical trials of the drugs are available to show the efficacy of the drugs and predict their clinical value; and (3) Vaccines urgently needed for responding to major public health emergencies or other vaccines recognized as urgently needed by the National Health Commission, where the benefits of the vaccines have been assessed to be greater than the risks. | (1) clinically urgent drugs in shortage, innovative and improved new drugs to prevent and treat major infectious diseases and rare diseases; (2) new varieties, dosage forms and specifications of medicines for children in line with the physiological characteristics of children; (3) vaccines and innovative vaccines needed for disease prevention and control; (4) drugs included in the breakthrough therapy drug procedure; (5) drugs in line with the conditional approval; (6) the NMPA stipulates that the other priority situations for review and approval. | In the emergence of public health emergency threats, as well as public health emergencies after the occurrence of public health emergencies, the NMPA can be decided by law on public health emergencies emergency prevention and treatment of drugs. |
| Application stage | Apply during the Phase I and II clinical trial phases, usually no later than before Phase III clinical trials are conducted. | Communication during clinical trials and pre-market application, application in NDA (no separate application required). | Communicating and exchanging information and applying in tandem with the NDA. | Before filing an application for registration. |
| Policy advantages | (1) Communication. The first communication within 6 months after inclusion shall be applied for according to the Class I meeting, and the communication in the critical phase of the clinical trial shall be applied for according to the Class II meeting; the first communication during the clinical trial, the meeting convened due to major safety issues/significant technical issues, the meeting of the clinical trial phase of the drug, and the consultation on general technical issues shall be prioritized.  (2) Development guidance. The applicant can submit the stage research information to the CDE, and the CDE will make comments or suggestions on the next step of the research program and provide feedback to the applicant.  (3) Priority review. The marketing authorization stage can be included in the priority review and approval procedure. | (1) Communication. This includes early communication on clinical research plans, key clinical trial designs, selection of efficacy indicators, conditions attached, and plans for involvement and implementation of post-marketing clinical trials.  (2) Accelerating marketing. Shorten the drug research and development process and accelerate the speed to market.  (3) Priority review. The marketing license stage can be included in the priority review and approval procedure. | (1) Shortening the timeframe. The time limit for the review of drug marketing registration is 130 days; the time limit for the review of rare disease drugs with urgent clinical needs that have been listed outside China but not in China is 70 days; the time limit for administrative approval has been shortened to 10 days.  (2) Optimizing the process. Verification, inspection, and approval of the common name of the drug is required to be prioritized, which requires the acceptance of the registration inspection within 2 days of the issuance of the inspection notification form, the need for registration verification, acceptance of the 25 days after the start, and in the review of the expiration of the time limit of 25 days before the completion of the verification and inspection work. Clinically needed rare disease drugs in the review time limit expire 15 days before completion.  (3) After communication and confirmation, additional technical information can be submitted. | (1) Shortening the timeframe. After the acceptance of the application for drug registration, the technical review of the registration declaration data will be carried out within 24 hours, and the on-site verification situation and relevant opinions will be reported to NMPA within 5 days.  (2) Optimize the process. The NMPA by the unified command, early intervention, rapid and efficient, scientific approval principle, the organization to speed up and synchronize the drug registration acceptance, review, verification, and inspection work. |
| Supporting Policies | Communication ( Class I meeting), Submission of Phase I study information (through communication), Prioritization of NDA for review and approval. | Conditional marketing approval based on surrogate endpoints, intermediate clinical endpoints, or early clinical trial data, Phase III clinical trial interim analysis data; NDA Priority Review Approval. | Reduction of review timeframe from 200 days to 130/70 days, priority verification, test approval of common names, rolling submission of information. | Early intervention, continuous follow-up, research and review linkage; shorter acceptance review and approval (acceptance 5D-24h, review 60D/200D-15D, approval 20D-3D) |
| Termination of proceedings | Terminate the breakthrough therapy drug procedure when the applicant or CDE finds that it no longer meets the conditions for inclusion. | If the conditions for conditional approval are no longer met, the procedure is terminated and the applicant studies the declaration by normal procedures. | When the conditions for priority review and approval are no longer met, review and approval will be conducted in accordance with normal procedures. | If the conditions for inclusion are no longer met, the special approval process for the drug shall be terminated. |

**Note:** The special approval procedure (SA) is mainly used for the review and approval of emergency drugs under public health emergencies, and the study on antineoplastic and immunomodulating agents in this paper does not involve this procedure.

**Supplementary** **Table3** Statistics on drug indications and their number

| Indication | Number of drugs |
| --- | --- |
| Non-small cell lung cancer | 33 |
| Lymphoma | 33 |
| Breast cancer | 16 |
| Multiple myeloma | 13 |
| leukaemia | 11 |
| Hepatocellular carcinoma | 10 |
| Melanoma | 8 |
| Ovarian cancer | 8 |
| Prostate cancer | 8 |
| Psoriasis | 8 |
| Rheumatoid arthritis | 7 |
| Other solid tumors | 6 |
| Metastatic colorectal cancer | 6 |
| Multiple sclerosis | 4 |
| Nasopharyngeal carcinoma | 3 |
| Systemic lupus erythematosus | 3 |
| Malignant pleural mesothelioma | 2 |
| Myelofibrosis | 2 |
| Myelodysplastic syndrome | 2 |
| Basal cell carcinoma | 2 |
| Medullary thyroid cancer | 2 |
| Ankylosing spondylitis | 2 |
| Neuroblastoma | 2 |
| Esophageal cancer | 2 |
| Ldiopathic pulmonary fibrosis | 2 |
| Atopic dermatitis | 2 |
| Head and neck squamous cell carcinoma | 2 |
| Gastric cancer | 2 |
| Gastrointestinal mesenchymal tumor | 2 |
| Sézary syndrome | 1 |
| Springtime keratoconjunctivitis | 1 |
| Bile duct cancer | 1 |
| Polyarticular juvenile idiopathic arthritis | 1 |
| Glioblastoma multiforme | 1 |
| Multicenter Castleman disease | 1 |
| Cervix | 1 |
| Thyroid cancer | 1 |
| Urothelial carcinoma | 1 |
| Neuroendocrine tumor | 1 |
| Renal cell carcinoma | 1 |
| Optic Nerve Myelitis Spectrum Disorders | 1 |
| Mycosis fungoides | 1 |
| Aplastic anemia | 1 |
| Paroxysmal sleep hemoglobinuria | 1 |
